# Point-of-care ultrasound of peripheral nerves in the diagnosis of Hansen's disease neuropathy

**DOI:** 10.3389/fmed.2022.985252

**Published:** 2022-09-09

**Authors:** Glauber Voltan, Fred Bernards Filho, Marcel Nani Leite, Natália Aparecida De Paula, Jaci Maria Santana, Claudia Maria Lincoln Silva, Josafá Gonçalves Barreto, Moises Batista Da Silva, Guilherme Conde, Claudio Guedes Salgado, Marco Andrey Cipriani Frade

**Affiliations:** ^1^Department of Interne Medicine - Dermatology, Faculty of Medicine of Ribeirão Preto, University of São Paulo, São Paulo, Brazil; ^2^Faculty of Medicine of Ribeirão Preto, University of São Paulo, São Paulo, Brazil; ^3^Federal University of Pará, Belém, Pará, Brazil

**Keywords:** leprosy, Hansen's disease, neuropathy, high-resolution ultrasound, cross-sectional area

## Abstract

**Introduction:**

Hansen's disease (HD) is the most common cause of treatable peripheral neuropathy in the world that may or may not involve skin manifestations, and physical examination based on simplified neurologic evaluation is a subjective and inaccurate procedure. High-resolution ultrasound (HRUS) can be used to evaluate peripheral nerves and is a validated technique of good reproducibility, permitting a detailed and precise examination.

**Objectives:**

We proposed to establish objective criteria for absolute values of the measurement of the CSA of peripheral nerves and their indices of the ΔCSA and ΔTpT in the diagnosis of Hansen's disease neuropathy as compared with healthy voluntaries.

**Materials and methods:**

In municipalities from different regions of Brazil, we randomly selected 234 volunteer Brazilian patients diagnosed with leprosy to be submitted to peripheral nerve echography and compared with 49 healthy Brazilian volunteers.

**Results:**

Hansen Disease assessed by high resolution ultrasound is a primarily neural disease that leads to multiple hypertrophic mononeuropathy characterized by CSA values exceeding normal limits (Med CT = 10.2 mm^2^; UT = 9.8 mm^2^; UPT = 9.3 mm^2^; CFFH = 18.3 mm^2^; T = 9.6 mm^2^), and the pattern of asymmetry (ΔCSA>2.5 mm^2^ with RR 13) and focality (ΔTPT > 2.5 mm^2^ with RR 6.4) of this thickening has higher sensitivity (76,1%) and specificity (87,8 %) for its early diagnosis that laboratory tests. Analyzing each subject, the percentage of thickened nerves detected among the total number of nerves assessed was higher among patients with HD than among healthy individuals (*p* < 0.0001). Individuals with two or more thickened nerves were at 24.1 times higher relative risk (95% CI: 6.74–88.98) of HD.

## Introduction

Hansen's disease (HD) is the most common cause of treatable peripheral neuropathy in the world. *Mycobacterium leprae* mainly resides in macrophages and Schwann cells, affecting myelination. It causes a primary neural disease involving the immunologic system (being induced or maintained by an immune response to the bacillus), and the neural damage results in disability and maintenance of stigmas. As proposed by the World Health Organization, nerve thickening is one of the cardinal signs for the diagnosis of a case of leprosy (1). When the first signals of nerve damage can be noted, at least 30% of the nerve fibers may be affected (2, 3). HD is a neural disease that may or may not involve skin manifestations (4–11). Cases of peripheral neuropathy accompanied by nerve thickening should lead clinicians to suspect a diagnosis of HD (12). Physical examination based on simplified neurologic evaluation including the palpation of peripheral nerves helps the diagnosis of nerve thickening and neuritis; however, this is a subjective procedure that may not detect the condition in very early cases, even by well-trained professionals (13).

High-resolution ultrasound (HRUS) can be used to evaluate most peripheral nerves and is a validated technique of good reproducibility, permitting a detailed and precise examination (14, 15). With HRUS, the peripheral nerves appear on a longitudinal view (cord-like pattern) as multiple tubular hypoechoic structures (dark gray bundles) intermingled with hyperechoic lines (perineurium—white), with their ensemble being covered with a hyperechoic line (epineurium). On a transversal view (honeycomb pattern), these structures are hypoechoic and round (fascicles) surrounded by hyperechoic bands (perineurium), and all of these fascicles are surrounded by a denser outer line (epineurium) (14, 15). HD has been characterized by greater peripheral nerve thickening defined by the increase in their cross-sectional area (CSA) (9, 10), usually asymmetrical (9), followed by other morphological changes, such as echogenicity and/or fascicular pattern; perineurium thickening; and peripheral nerve vascularization that can be detected by intraneural and/or perineural Doppler signals (16, 17). In addition to parameters regarding the absolute values of CSA measurements, Frade et al. (10), among others, have suggested the measurement of the asymmetry index [ΔCSA = (>right or left CSA) – (< right or left CSA)] in the evaluation of leprosy and have demonstrated that this index is highly sensitive and specific for the differentiation between the nerves of healthy individuals and the nerves of patients with leprosy. Regardless of the classification as multibacillary or paucibacillary, the fusiform thickening of the ulnar nerve starts in the ulnar sulcus and reaches a maximum of 4 cm above the medial epicondyle (9, 10, 18–20). However, Frade et al. (10) have pointed out that the difference between the measurement of the thickened ulnar nerve in the distal region of the arm and that in the elbow region, or vice versa, had more than 90% specificity for leprosy. This characteristic finding may be of help, especially in the diagnosis of primary or pure neural leprosy (PNL), in which the skin lesions are absent and in the differentiation between leprosy and other neuropathies, in which a diffuse nerve increase may occur.

So, we proposed to establish the objective criteria of the measurements of absolute values of the CSAs of the peripheral nerves and their indices of the asymmetry and focality in the diagnosis of Hansen's disease neuropathy as compared with healthy voluntaries.

## Ethical considerations

The present study was approved by the Research Ethics Committee of the University Hospital, Faculty of Medicine of Ribeirão Preto, University of São Paulo (protocol no. 2.165.032, MH-Brasil and 92228318.1.0000.5440). Written informed consent was obtained from all participants, including the parents/guardians of subjects younger than 18 years. All procedures involving human beings followed the ethical standards of the Declaration of Helsinki (1975/2008).

## Casuistry

Municipalities of different regions of Brazil (north, northeast, and southwest) whose health professionals had been habilitated by the National Reference Center for Sanitary Dermatology with emphasis on leprosy of HCFMRP-USP were selected between 2016 and 2020. In these regions, we randomly selected 234 volunteer Brazilian patients diagnosed with leprosy by these teams to be submitted to peripheral nerve echography. As a comparative (control) sample of healthy Brazilian volunteers, we used cases of our service, as reported by Voltan et al. (2022) (Annex 1); to carry out this work, we obtained a sample number of 97 for the Brazilian population of 211 million inhabitants, with a confidence level of 95% and a margin of error of 10%. Our sample consisted of 66 individuals; however, since the distribution of the CSA of the right and left nerves was homogeneous, 132 neural points were considered for statistical analysis, being the largest sample among the evaluated studies and fulfilling the sample calculation criteria for our population. Calculation performed with https://calculareconverter.com.br/calculo-amostral/ and verified at https://solvis.com.br/calculos-de-amostragem/ for 211 million and 132 neural points of 66 individuals with 95% CI have a margin of error of up to 8.53%. In addition, we included in the same data bank unpublished data on the 18 tibial nerves of nine individuals aged 15–60 years (mean: 7.6 ± 0.94 mm^2^; median: 8.0 mm^2^).

## Clinical evaluation

Clinical evaluation was performed by dermatologists and HD specialists habilitated by the program of the Health Ministry. The teams were not involved in the capture and HRUS analysis of the images.

## Peripheral nerve echography

**Figure 1:**
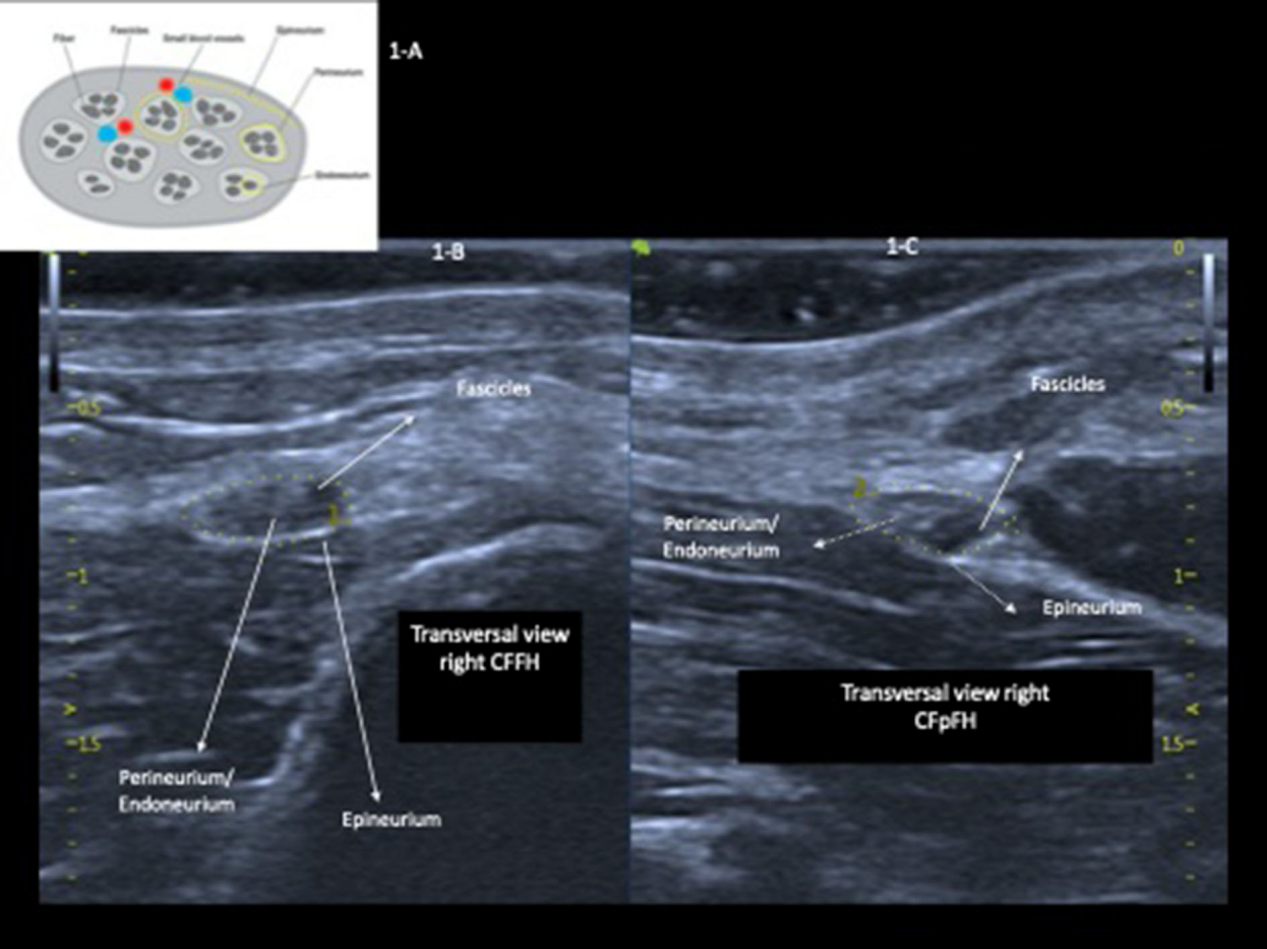
Drawing of a cross-section of a peripheral nerve **(A)**; high-resolution echography of the ulnar nerve in the right (RPT) and the left (LPT) cubital pretunnel with preserved cross-sectional area, echogenicity, and fascicular pattern **(B,C)**.

The general portable ultrasound devices used were Mindray M5, Samsung HM70-A, and Vinno6, equipped with high-frequency transducers, with frequencies ranging from 4 to 17 MHz. Each nerve was scanned on crosswise and lengthwise sections, and the cross-sectional area (CSA) of the transversal sections was obtained with adjustment of the angle perpendicular to the insonated nerve surface, without pressure on the structures. The neural points assessed were defined according to their proximity to anatomical bone references, facilitating the reproducibility of the method by being well-established sites for neural compression or more common sites of electrophysiological assessment. The CSA was determined at these sites with a continuous trace, internally to the hyperechoic borders of the epineurium (Figures 1A,B). For comparison with the literature, all patients were submitted to already established echographic assessment of 10 neural sites, that is, median nerves in the carpal tunnel (between the scaphoid and pisiform bones); ulnar nerves in the cubital tunnel (between the medial epicondyle and olecranon) and in the distal third of the arm (cubital pretunnel−3–5 cm above the medial epicondyle); the common fibular nerves in the fibular head topography; and the tibial nerves in the tarsal tunnel, all of them bilaterally. As an exception, four new additional sites were established for the assessment of routine focality, that is, common fibular nerves in the thigh (pre-fibula head or distal third of the thigh between 3 and 8 cm proximal to the fibula head) and median nerves in the distal third of the forearm (3–5 cm proximal to the carpal tunnel). The nerves of the upper limbs were assessed with the patient sitting and with elbows flexed 60–90°, while the nerves of the lower limbs were assessed with the patient sitting or in the dorsal decubitus with legs slightly flexed 90–130°.

All data collection procedures were carried out during active search campaigns in different regions of Brazil. After 2 or 3 months of collection, the same researcher opened the images on his personal computer and analyzed each image, extracting only objective data from the CSA for statistical analysis. All other results were obtained using mathematical formulas performed in Excel to arrive at ΔCSA and ΔTpT. In short, the researcher's experience helped in carrying out the measurements, but as they are objective data, if any other researcher performs them following the same standards, they should have the same results.

## Statistical analysis

Excel^®^ software was used to transpose the CSA values (mm^2^) obtained from photographs of the ultrasound examination, and Prism 8 software for macOS was used for later data analysis. The means, standard deviations, and median absolute CSA values were calculated for each of the 14 nerve sites assessed (seven on the right side and seven on the left side). The paired *t*-test was used to analyze asymmetries. We calculated the indices (Δ) of the differences of absolute CSA values between the right and the left side (ΔCSAs) and of the differences between the absolute CSA values in the same nerve. The neural sites were considered to be thickened when their CSA values, extracted from the database from Voltan et al. (2022), were higher than the reference values for normal individuals summed to two times the values of the standard deviations (SDs). Similarly, each neural site was considered to be asymmetrical when the ΔCSA was higher than the reference values plus two times the SD for normal individuals. The same reasoning was used for the ΔTPT to consider the focality. The total number of thickened/altered neural sites was counted using the “cont.se” function of Excel software, and the total number of altered sites in terms of asymmetry (ΔCSA) and focality (ΔTPT) was calculated for in each one.

The total number of thickened neural sites was calculated for each individual using the “cont.se” function of Excel. Similarly, each neural site was considered to be asymmetrical or with focality when the ΔCSA and ΔTPT, respectively, were higher than RV + 2 SD. The total number of altered sites in terms of asymmetry (ΔCSA) and focality (ΔTPT) was calculated using the “cont.se” function of Excel. In order to assess the discriminatory power for the diagnosis of Hansen's neuropathy by ultrasound, the ROC curve was applied to the number of altered sites according to CSA, ΔCSA, and ΔTPT as compared with the values obtained for healthy individuals.

## Results

The clinical–demographic characteristics and the anti-PGL1 results of the population sample studied are given in Table 1.

**Table 1:** Distribution of the population sample by sex, age range, state of origin, operational classification, and anti-PGL 1 data.

**Variables**		***N***	**%**
Sex	Male	113	48.29
	Female	121	51.71
Age (years)	4–14	38	16.24
	15–60	176	75.21
	60–81	20	8.55
Region of origin.	North	72	30.77
	Northeast	67	28.63
	Southeast	95	40.60
Classification of Hansen's disease	DD	159	67.95
	DT	36	15.38
	DV	18	7.69
	I	5	2.14
	PN	5	2.14
	T	4	1.71
	V	7	2.99
Anti-PGL 1	Negative (0.47 ± 0.32)	86	36.75
	Positive (2.85 ± 3.96)	64	27.35
	Not determined	84	35.90
Mean Anti-PGL 1 index	1.49 ± 2.85	150	64.10

DD, dimorphic–dimorphic; HDT, dimorphic–tuberculoid; DV, dimorphic–Virchowian; I, indeterminate; PN, pure neural; T, tuberculoid; VL, virchowian.

The values detected for the patients with HD were compared with the normal CSA patterns of healthy Brazilian individuals based on the values established by Voltan et al. (2022).

## Absolute CSA values (mm^2^) of the peripheral nerves and their indices

The 234 patients were assessed by bilateral HRUS of the peripheral nerves: median in the carpal tunnel (Med CT), ulnar in the cubital tunnel (UT) and cubital pretunnel (UPT), common fibular in the fibula head (CFFH), and tibial in the medial malleolus (T). Based on initial observations during fieldwork, new sites were routinely established as follows: median nerve in the forearm (Med FA) and the common fibular nerve in the thigh proximal to the fibula head (CFTH), and for this reason, there was a variation of the sample (n), with measurements of Med TC being obtained for 100 (43 %) patients and measurements of CFTH being obtained for 151 (64.5 %) patients.

The mean, standard deviation, and median values of the absolute CSA values in mm^2^ and of the differences between the right and left sides (ΔCSA) and between sites of the same nerve (ΔTPT) are presented in Table 2 according to age range.

**Table 2:** Distribution of ultrasound measurements (CSA, ΔCSA, and ΔTPT) according to age range, total sample, and upper limit.

**Variables**	**Age range (years)**	**4–14**	**15–30**	**31–45**	**46–60**	**62–81**	**Sample**	**Upper limit (HVs)**
							**(15–60 years)**	**(mean + 2SD)**
	(*n*) Men	14	24	36	25	14	85	–
	(*n*) Women	24	38	29	24	6	91	–
	(*n*) Total (Right + Left)	76	124	130	98	40	352	–
	Mean ± SD [median]	10.3 ± 2.8 [11]	22,3 ±4,9 [22]	37.0 ± 4.0 [37]	52.2 ± 4.2 [52]	69.9 ± 5.2 [69]	36.1 ± 12.6 [36]	–
	Site	Mean ± SD [median]
Peripheral nerves	Med CT	7.8 ± 2.2 [7]	10.1 ± 2.8 [10]	11.0 ± 2.8 [10.9]	12.1 ± 3.2 [12]	11.3 ± 3.3 [11]	11.0 ± 3.0 [10.7]	10.2
	UT	5.7 ± 1.8 [5]	7.8 ± 3.1 [7]	8.3 ± 3.0 [8]	9.1 ± 3.2 [9]	10.8 ± 4.1 [10]	8.3 ± 3.2 [7.9]	9.8
	UPT	5.3 ± 1.8 [5]	7.9 ± 4.9 [7]	9.3 ± 6.9 [7]	9.7 ± 4.5 [8.5]	11.4 ± 7.3 [9.4]	8.9 ± 5.7 [7]	9.3
	CFFH	10.4 ± 3.9 [10]	15.9 ± 5.2 [15]	18.9 ± 6.4 [18]	21.2 ± 7.0 [20.7]	19.5 ± 6.5 [17.8]	18.4 ± 6.5 [17]	18.3
	T	6.8 ± 2.1 [7]	9.2 ± 4.2 [9]	11.5 ± 4.9 [10.6]	12.8 ± 5.0 [11.9]	14.0 ± 7.0 [11.8]	11.0 ± 4.9 [10]	9.6
ΔCSA (mm^2^)	Med CT	1.3 ±1.3 [1.0]	1.7 ± 1.6 [1.0]	1.5 ± 1.3 [1]	2.6 ± 2.1 [2]	2.8 ± 2.0 [2.6]	1.9 ± 1.8 [1.3]	2.2
	UT	1.1 ± 1.2 [1]	1.8 ± 1.7 [1]	1.9 ± 1.8 [1.2]	1.8 ± 1.5 [1.9]	1.7 ± 2.3 [1]	1.9 ± 1.7 [1.2]	3.1
	UPT	1.0 ± 0.8 [1]	1.6 ± 1.8 [1]	2.5 ± 5.0 [1.1]	2.5 ± 4.3 [1]	2.8 ± 2.6 [2]	2.2 ± 3.9 [1.0]	1.4
	CFFH	1.5 ± 1.5 [1]	2.3 ± 2.5 [1.3]	3.6 ± 5.9 [2]	3.8 ± 3.7 [3]	4.2 ± 3.6 [4.3]	3.2 ± 4.4 [2]	2.3
	T	1.1 ± 1.2 [1]	1.4 ± 1.6 [1]	2.3 ± 3.0 [2.0]	2.9 ± 3.1 [2.0]	3.8 ± 3.0 [2.4]	2.1 ± 2.7 [1.1]	–
ΔTPT (mm^2^)	Ulnar (UT and UPT)	0.9 ± 1.1 [1]	1.8 ± 2.9 [1]	2.7± 5.1 [1]	2.2 ± 3.2 [1.3]	3.3 ± 4.5 [1.8]	2.2 ± 3.9 [1]	2.6
								
Analysis of new neural sites
	Total *n* = 100	16	34	62	62	26	158	–
Peripheral nerves	Med FA	6.4 ± 2.0 [6.7]	9.5 ± 3.7 [8.3]	9.9 ± 3.4 [9.0]	9.8 ± 2.4 [9.9]	13.0 ± 12.3 [10.4]	9.8 ± 3.1 [9.3]	–
ΔCSA (mm^2^)	Med FA	1.6 ± 1.1 [1.5]	1.9 ± 1.9 [1.6]	2.2 ± 2.0 [1.4]	1.5 ± 1.5 [1.2]	5.8 ± 13 [3.1]	1.9 ± 1.8 [1.3]	–
ΔTPT (mm^2^)	Median (CT and FA)	1.5 ± 1.0 [1.4]	2.4 ± 2.0 [1.7]	2.5 ± 2.0 [1.8]	2.9 ± 2.4 [2.0]	5.4 ± 9.6 [3.2]	2.6 ± 2.2 [1.9]	–
	Total n = 151	22	68	102	80	30	250	–
Pripheral nerve	CFTH	9.3 ± 3.2 [8.9]	14.5 ± 4.7 [13.9]	17.8 ± 6.8 [16]	18.8 ± 5.2 [18.5]	18.3 ± 6.8 [17.3]	17.2 ± 6.0 [16]	–
ΔCSA (mm2)	CFTH	1.2 ± 1.4 [0.6]	1.8 ± 1.6 [1.6]	4.3 ± 4.7 [3.5]	3.8 ± 3.8 [3.0]	5.8 ±4.3 [4.7]	3.4 ± 3.9 [2.2]	–
ΔTPT (mm^2^)	Fibular (CF and TH)	1.2 ± 0.9 [1]	2.6 ± 2.6 [2]	3.6 ± 3.4 [2.4]	3.5 ± 2.6 [2.8]	3.5 ± 2.9 [2.8]	3.3 ± 2.9 [2.4]	–

(n), number; Med, median; CT, carpal tunnel; UT, ulnar tunnel; UPT, ulnar pretunnel; CFFH, common fibular in the fibula head; T, tibial; FA, forearm; CFTH, common fibular thigh; ΔCSA, difference between the right and left nerves at the same site of assessment; ΔTPT, difference between the same nerve at different sites. Data are reported as mean ± standard deviation, and mean (mm^2^).

## Assessment of CSA values according to gender

**Figure 2:**
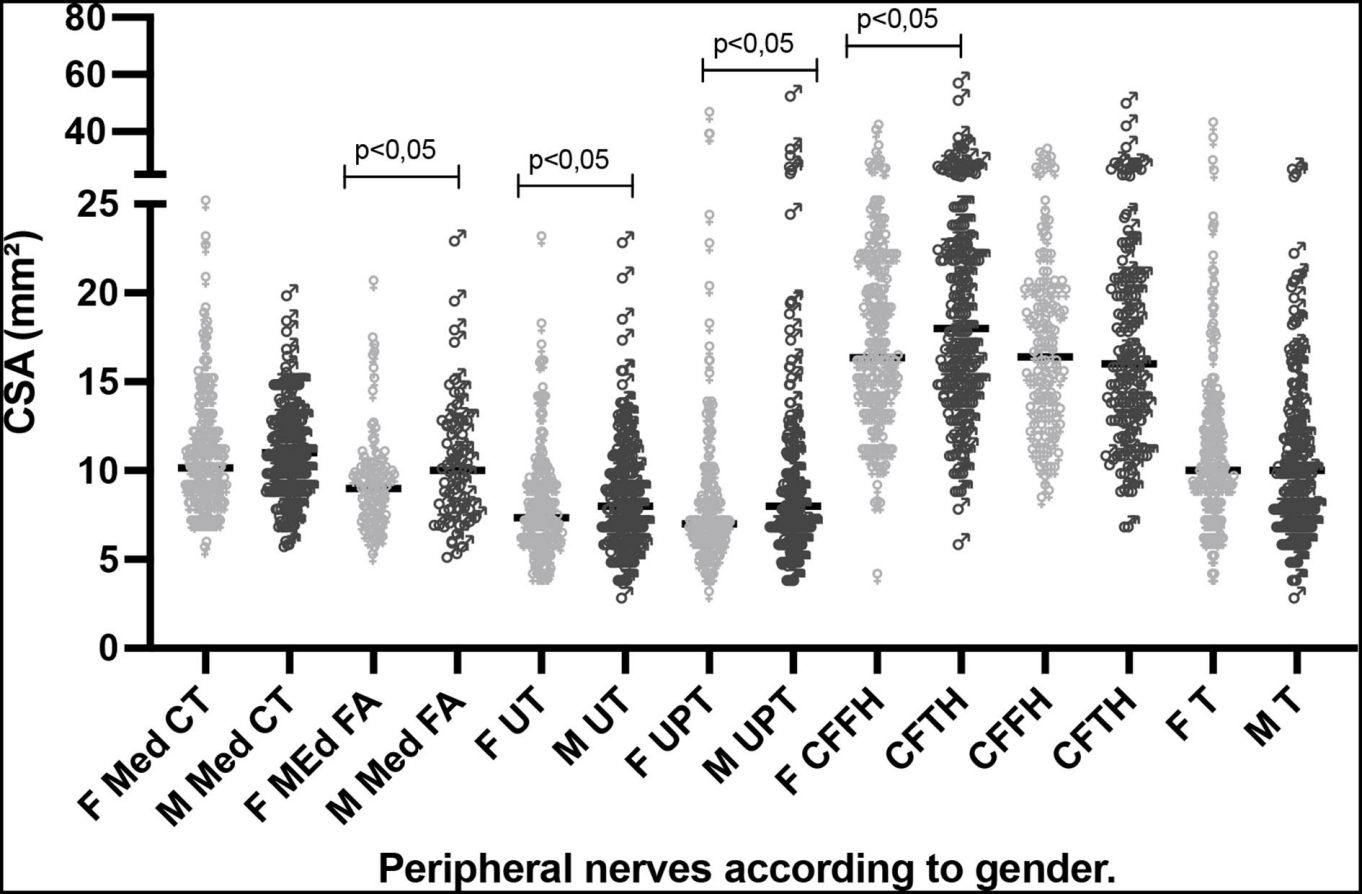
Distribution of CSA (mm^2^) values at neural sites according to male and female gender for the age range of 15–60 years. F, female; M, male; CSA, cross-sectional area; Med CT, median carpal tunnel; Med FA, median forearm; UT, cubital ulnar tunnel; UPT, cubital ulnar pretunnel; CFFH, common fibular head of the fibula; CFTH, common fibular thigh; T, tibial.

The absolute peripheral nerve CSA values (age range: 15–60 years) divided into male and female gender tended to be higher for men, with a difference at the sites of the Med FA, UT, UPT, and CFFH nerves (Figure 2).

## Assessment of CSA values according to age range

**Figure 3:**
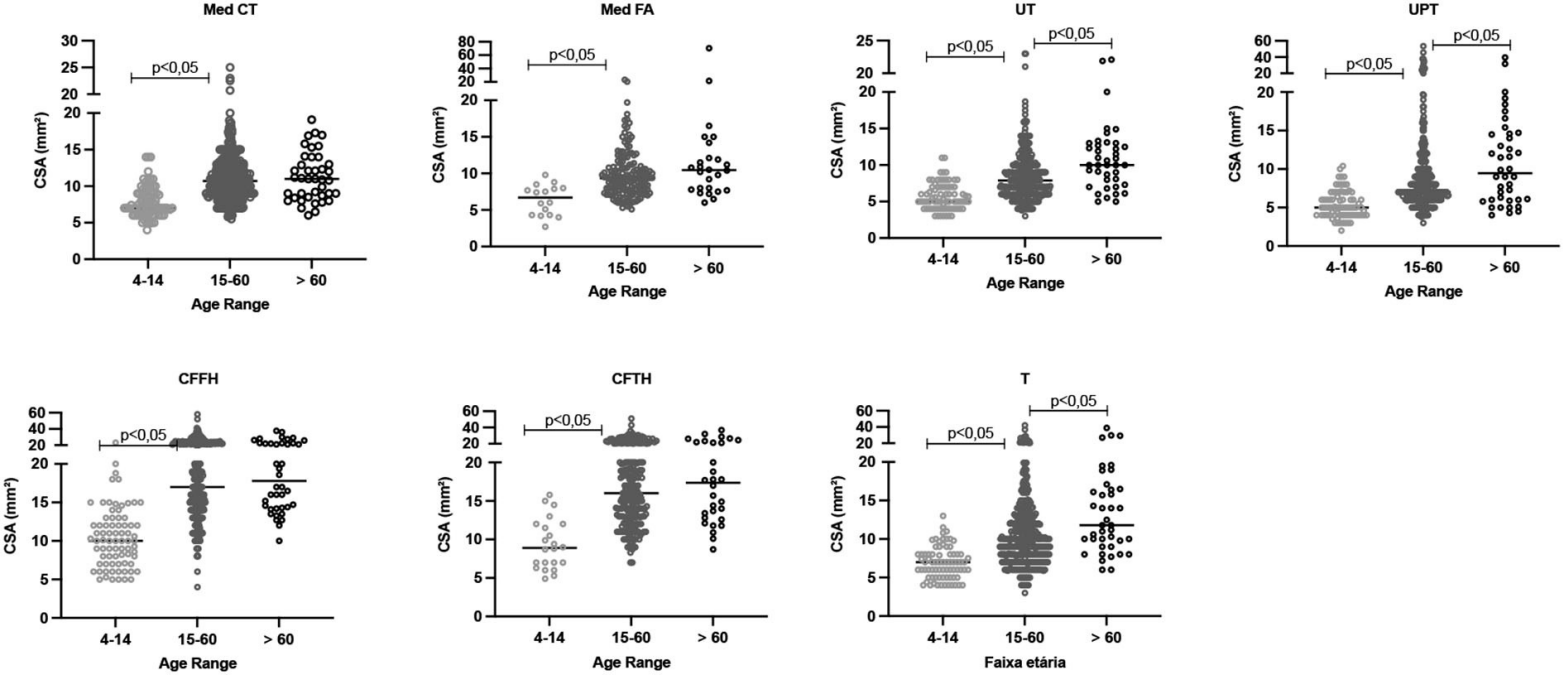
Distribution of absolute CSA (mm^2^) values for the neural sites assessed in patients with leprosy according to age range, with differences highlighted (*p* < 0.05). CSA, cross-sectional area; Med CT, median carpal tunnel; Med FA, median forearm; UT, cubital ulnar tunnel; UPT, cubital ulnar pretunnel; CFFH, common fibular in the fibula head; CFTH, common fibular thigh; T, tibial.

We observed a gradual increase in mean and median peripheral nerve CSAs with increasing age range in patients diagnosed with Hansen's disease. There was a significant difference between the age-group of 0–14 years and the remaining groups (p < 0.05). Among the age-groups of 15–60 and > 60 years, this increase occurred only for the UT, UPT, and tibial sites, as shown in Table 2 and Figure 3.

**Figure 4:**
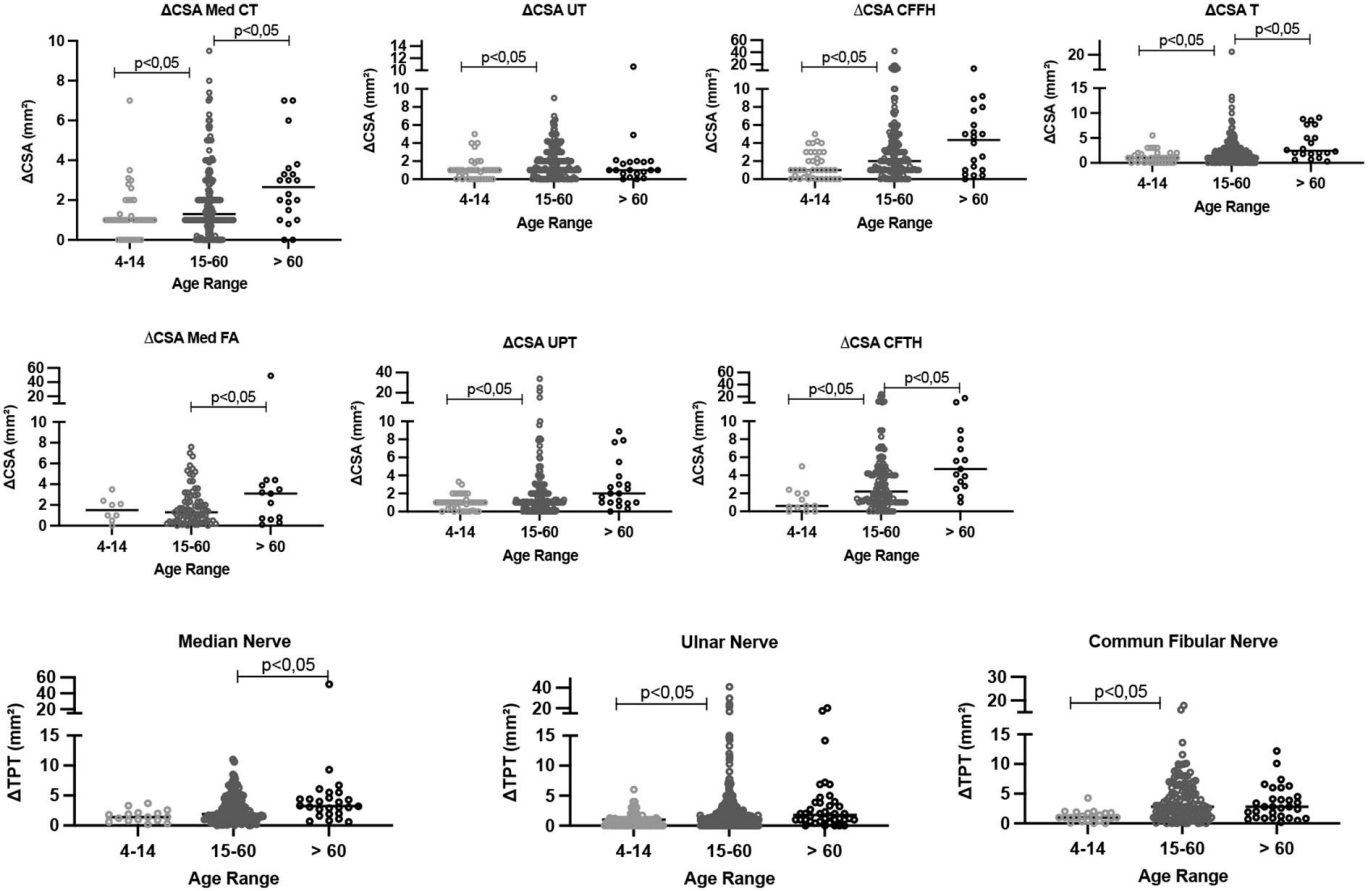
Comparative analysis of the difference between the right and left CSA values (ΔCSA) and the values in the same nerve (ΔTPT) according to age range. CSA, cross-sectional area; Med CT, median carpal tunnel; Med FA, median forearm; UT, cubital ulnar tunnel; UPT, cubital ulnar pretunnel; CFFH, common fibular in the fibula head; CFTH, common fibular thigh; T, tibial. ΔCSA, difference between the right and the left nerve at the same site of assessment, ΔTPT, difference between different sites of the same nerve.

As observed for the absolute values, the ΔCSA and ΔTPT values differed between the age-group of 0–14 years and the remaining groups (*p* < 0.05), except for the Med FA site. Comparison of the age-groups of 15–60 and > 60 years revealed that the differences (Δ) were greater in the age-group of >60 years at the Med CT, Med FA, CFTH, and T sites, as shown in Table 2 and Figure 4.

## Comparative analysis of healthy individuals and patients with leprosy

We compared 49 healthy individuals (98 neural sites) with 176 individuals diagnosed with leprosy (352 neural sites) in the age range of 15–60 years.

Since these were not paired samples and did not have parametric distribution, we compared both the absolute CSA values of peripheral nerves and the differences between them using the Mann–Whitney test.

**Figure 5:**
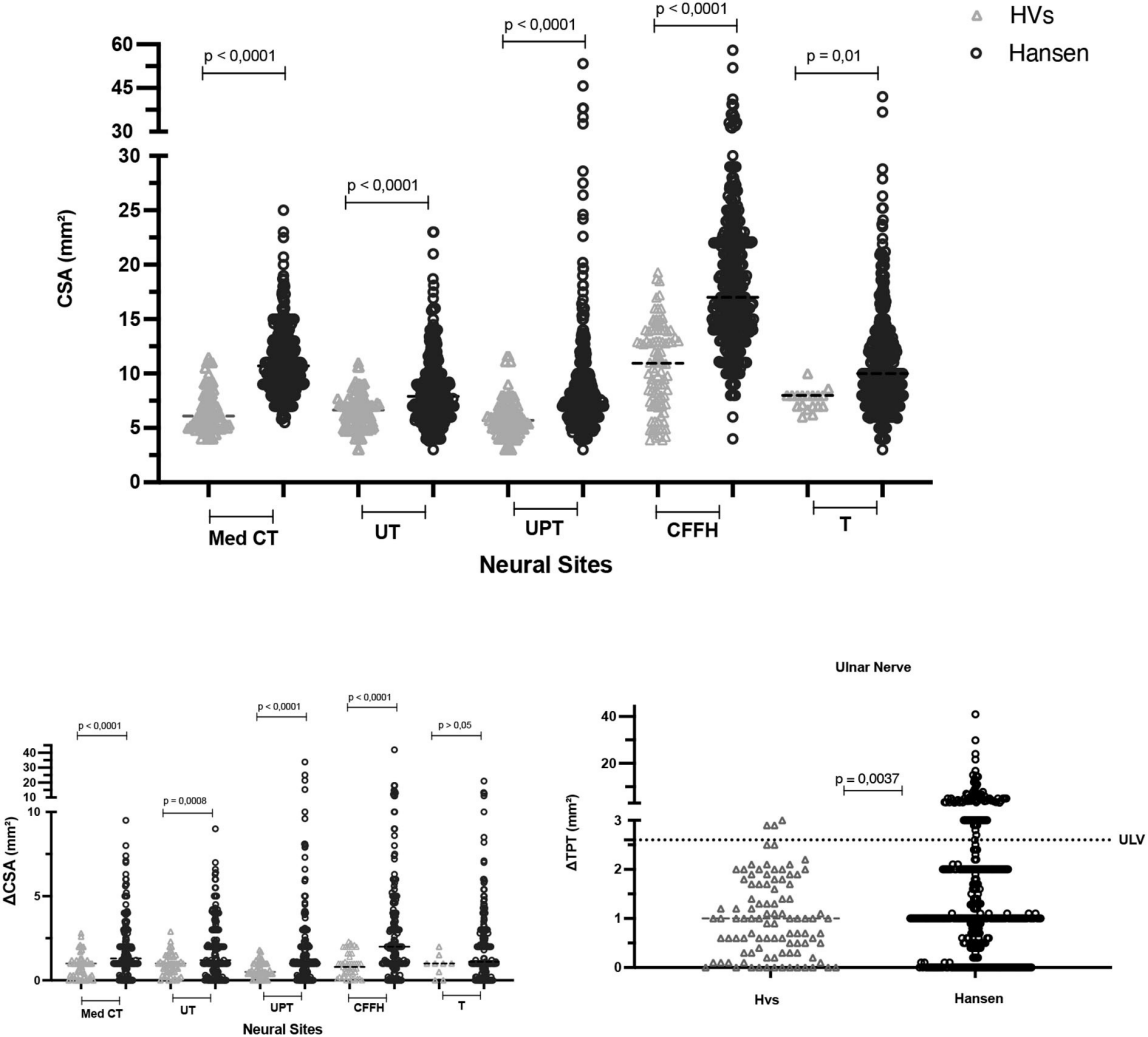
Comparative analysis of peripheral nerve CSA, ΔCSA (mm^2^), and ΔTPT (mm^2^) values between healthy individuals and patients with Hansen's disease aged 15–60 years. CSA, cross-sectional area; Med CT, median carpal tunnel; Med FA, median forearm; UT, cubital ulnar tunnel; UPT, cubital ulnar pretunnel; CFFH, common fibular in the fibula head; CFpFH, common fibular proximal fibular head; T, tibial; ΔCSA, difference between the right and the left nerve at the same site of assessment; ΔTPT, difference between the same nerve at different sites of assessment; HVs, healthy volunteers; Hansen, patients with Hansen's disease.

The results revealed higher median and mean absolute CSA values of peripheral nerves among the patients with leprosy compared with healthy individuals at all possible comparison sites, as illustrated in detail in Figure 5.

The mean absolute CSA value of the tibial nerves of patients was 11.0 ± 4.9 and was higher than the value detected in healthy individuals, as also observed by Voltan et al. (unpublished data) who obtained a mean value of 7.6 ± 0.94 mm^2^ and a median of 8.0 mm^2^, and as reported by Kerasnoudis et al. (21) (6.36 ± 1.4), Tagliafico et al. (22) (9.6 ± 4), Boehm et al. (23) (9.1 ± 2.2), and Grimm et al. (24) (10.2 ± 2.0).

**Figure 6:**
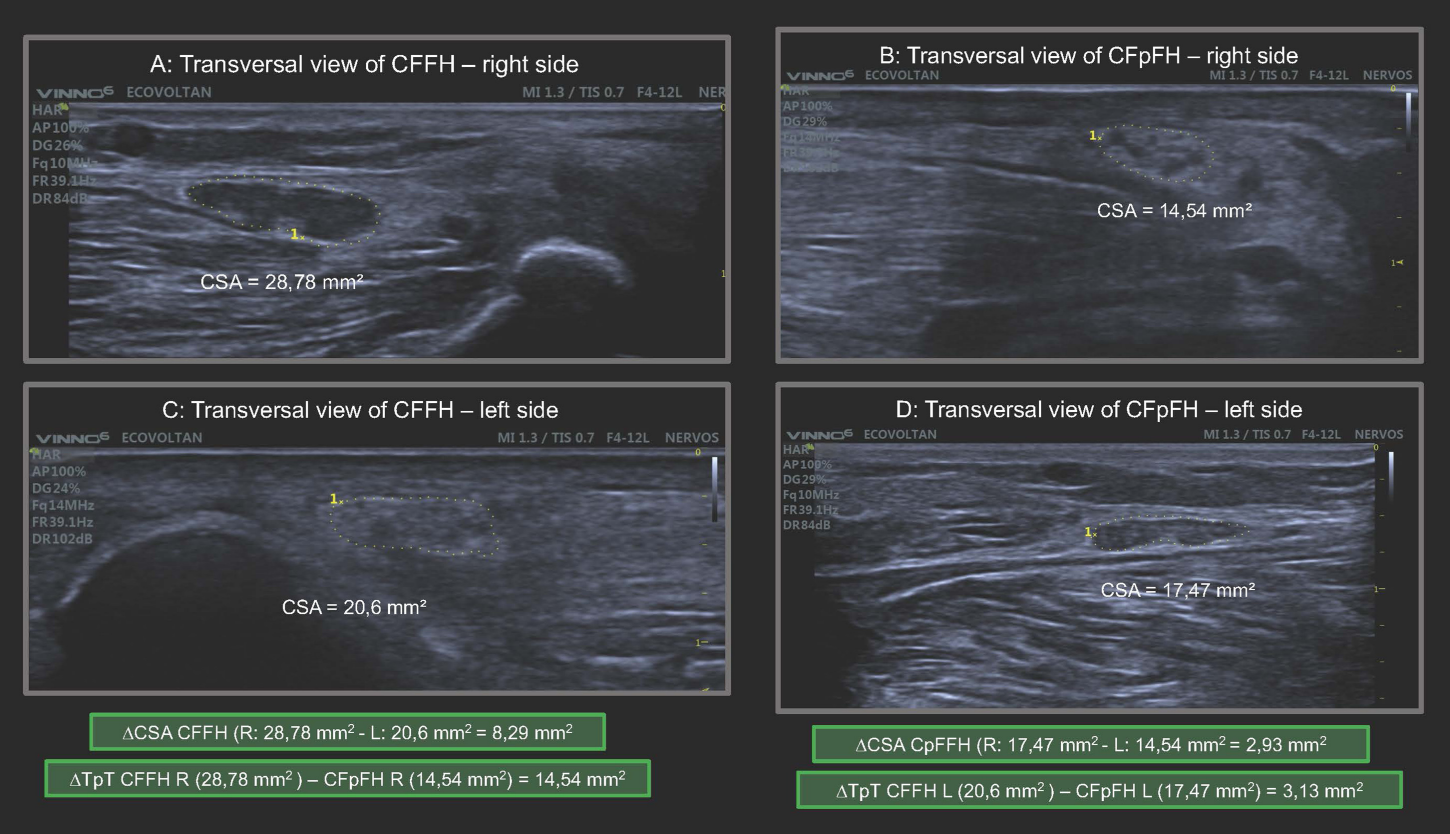
Transversal view of common fibular nerve on the right and left sides in the same HD patient demonstrating enlargement of peripheral nerve, asymmetry (ΔCSA) and focality (ΔTpT) major than normal references values. CSA, cross-sectional área; CFFH, common fibular nerve at fibular head; CFpFH, common fibular nerve proximal at fibular head; R, right; L, left; ΔCSA, asymmetry; ΔTpT, focality. In the green frame we highlight the calculation of ΔCSA and ΔTpT.

Regarding the asymmetry of CSA values at each neural site assessed as calculated by the respective differences between sides (ΔCSA), we observed higher mean and median differences in the group of patients with leprosy than in the group of healthy individuals at all neural sites, except for the tibial nerve, which showed no difference. The mean and median values of focality, measured by the difference between two sites in the same nerve (ΔTPT) for the ulnar tunnel/ pretunnel nerve, were also higher in the group of patients with leprosy than in healthy individuals (Figures 5, 6).

## Analysis of the absolute number and percentage of thickened nerves per individual

**Figure 7:**
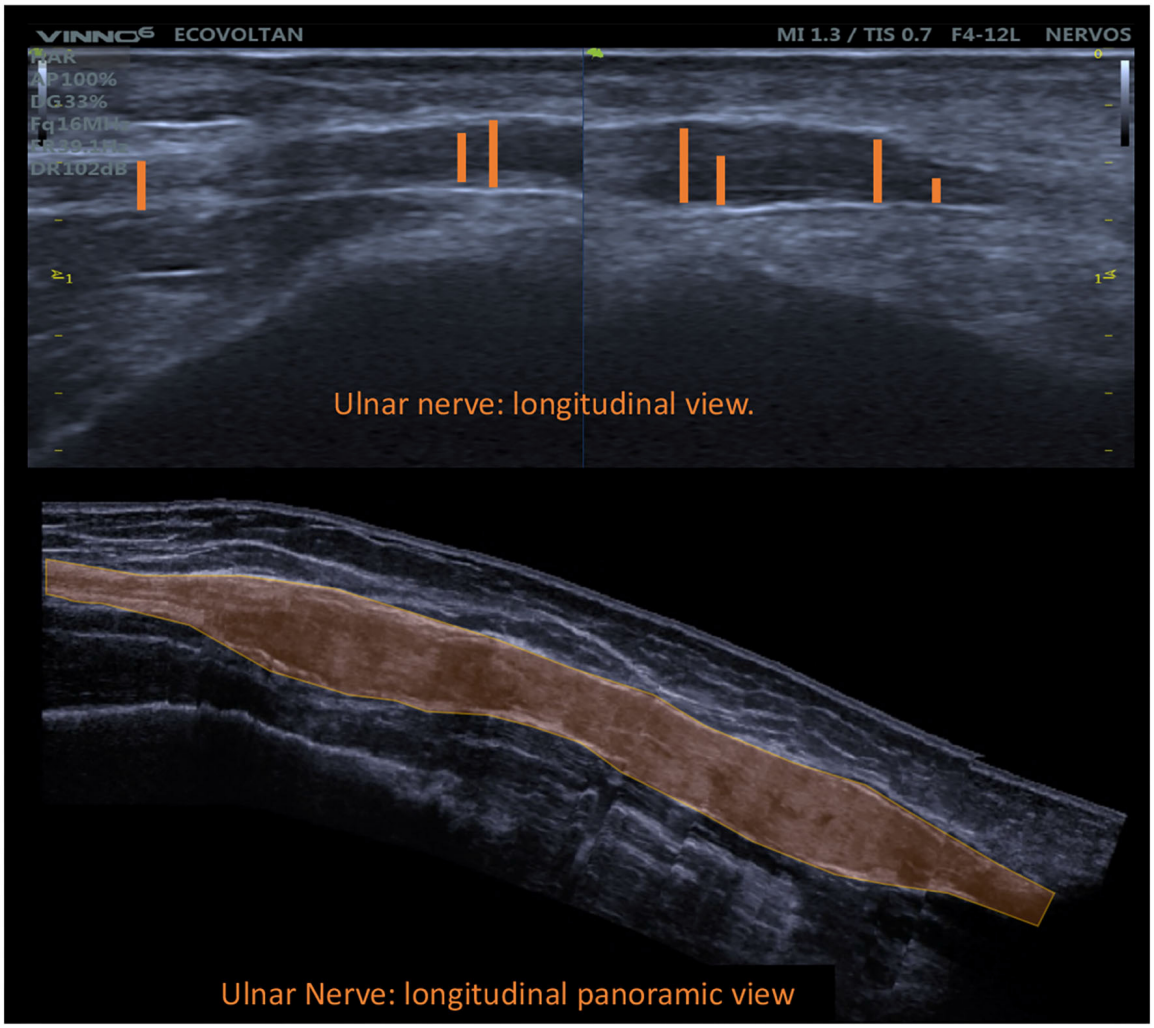
Longitudinal view of the common fibular nerve with fusiform enlargement demonstrating focality (ΔTpT).

Analyzing each subject, the percentage of thickened nerves detected among the total number of nerves assessed was higher among patients with HD than among healthy individuals (*p* < 0.0001). The ROC curve revealed an AUC of 89.1 (95% CI: 84.6–93.5%, *p* < 0.0001) (Figure 7). These findings indicate that when the percentage of thickened nerves was higher than 16.5% (more than two altered nerve sites), the sensitivity reached 76.1% (CI: 69.3–81.8) and specificity reached 87.8% (75.8–94.3), with a relative risk for HD of 6.2.

Considering the 16.5% sensitivity detected, for a binomial analysis, we divided our sample into subjects with up to two nerves altered ( ≤ 2) and individuals with more than two nerves altered (>2). The chi-square value with Yates correction was 52.03 (*p* < 0.00001), with individuals having two or more thickened nerves at 24.1 times higher relative risk (95% CI: 6.74–88.98) of Hansen's disease.

## Analysis by number of asymmetrical sites per subject

**Figure 8:**
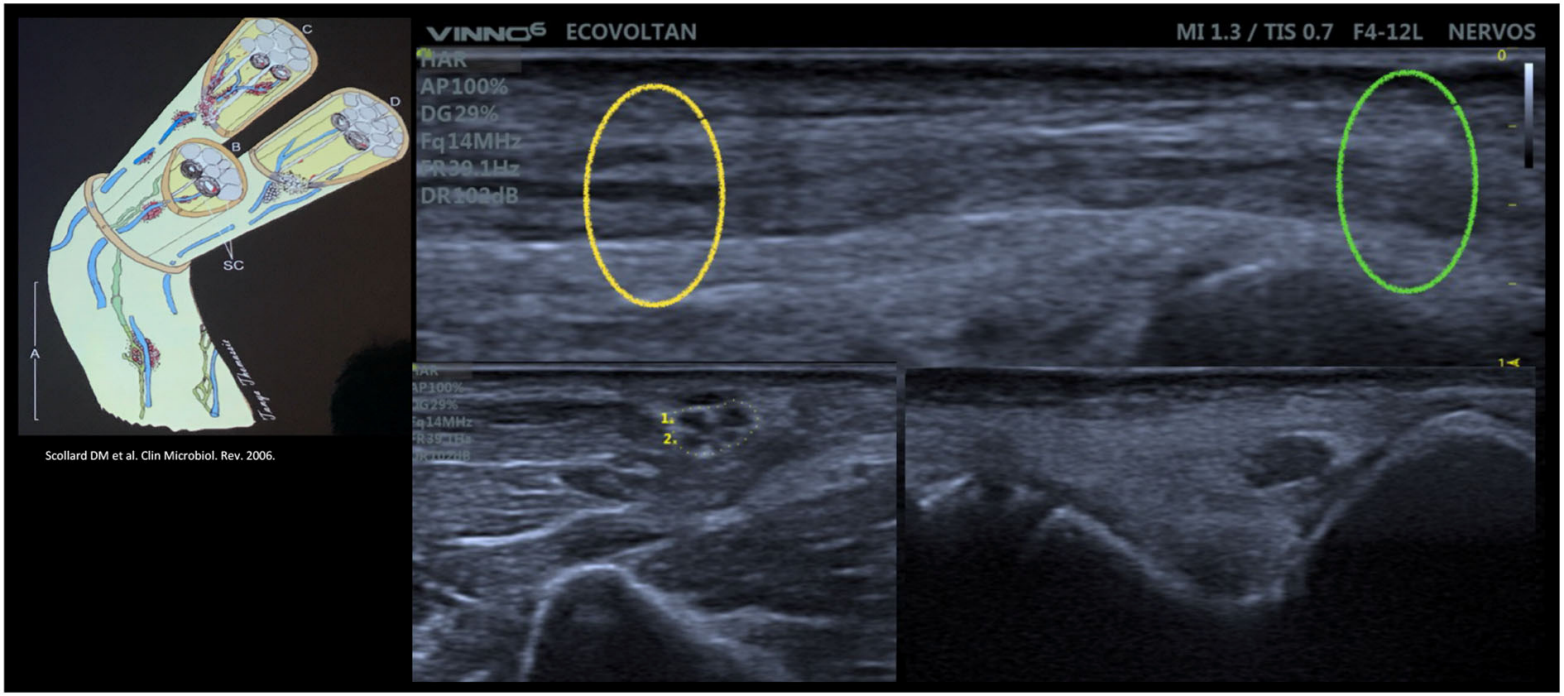
Intraneural focality pattern [Scollard pattern (4) demonstrated by Voltan, G and Frade, MAC with the high-resolution ultrasound] of the ulnar nerve on the longitudinal view (up) and transversal view (down right and down left) showing the different fascicle patterns in the same nerve.

Analysis of asymmetries among the neural sites (ΔCSA) defined as altered (more than RV ± 2 SD) per individual by the ROC curve when comparing healthy individuals and patients with HD revealed that the area under the curve was 85.09 (95% CI: 0.79–0.90, *p* < 0.0001), as demonstrated in Figure 8. Our data showed 79% sensitivity (72.37–84.35) and 87.8% specificity (75, 76–94, 27) when more than 10% of the neural sites evaluated presented altered ΔCSA (>2.5 mm^2^), with a relative risk of 6.45 for HD.

Considering the 10% sensitivity index detected, for a binomial analysis, and dividing our sample into individuals who had zero nerves altered in terms of asymmetry and individuals with one or more altered asymmetries (≥1), the chi-square value with Yates correction was 71.6 (*p* < 0.0001), and individuals with one or more asymmetrical nerves were found to be at 13 times higher relative risk (95% CI: 5.98–28.79) of HD.

## Analysis by the difference between two sites of the same nerve per individual (Focality)

**Figure 9:**
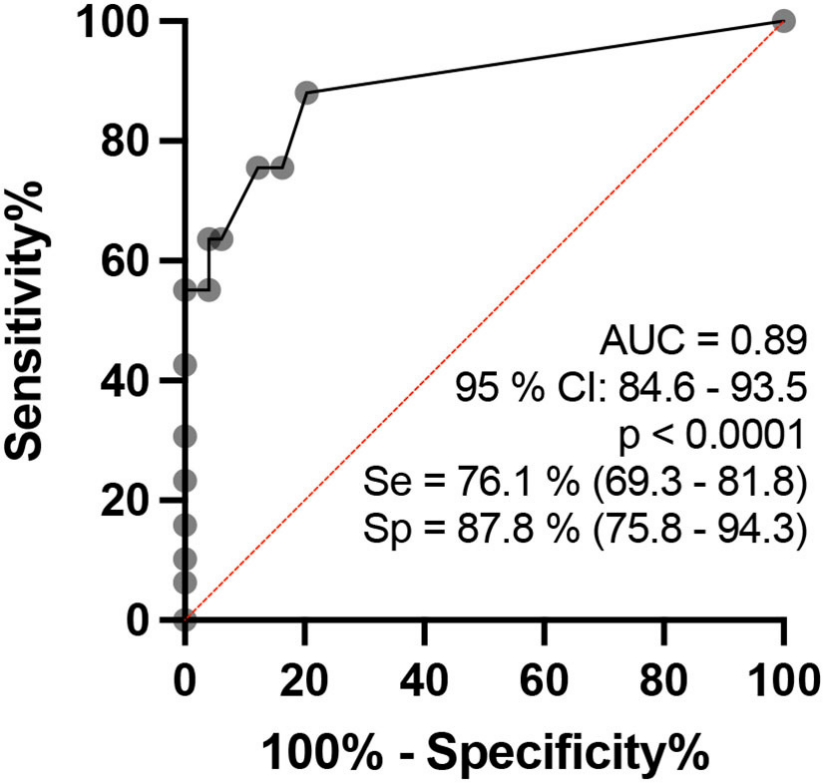
ROC curve analysis of the percentage of altered nerves among the nerves assessed per individual in healthy and HD groups.

When comparing healthy individuals with patients with HD, we checked the cutoff point for the number of altered ΔTpT per individual, and we found no difference between groups (*p* > 0.05) (Table 3, Figure 9).

**Table 3:** ROC curve table comparing the percentage of nerves with at least one altered ΔTpT (>2.5 mm^2^ - focality) between healthy individuals and patients with leprosy.

	**Sensitivity%**	**95% CI**	**Specificity%**	**95% CI**	**Likelihood**
					**ratio**
>0.5000	39.77	32.83–47.15%	93.88	83.48–97.90%	6.496
>1.500	8.523	5.233–13.58%	100.0	92.73–100.0%	

For a binomial analysis, we divided our sample into individuals who had no altered ΔTPT and individuals with one or more altered ΔTPT (≥1). The chi-square value with Yates correction was 18.3 (*p* < 0.0001), and individuals with one or more altered ΔTPT were found to have a 6.4 higher relative risk (95% CI: 2.5–21.9) of HD.

## Discussion

Over the last decade, HRUS has become an invaluable diagnostic method for the assessment of peripheral nerves, being used for the assessment of focal and diffuse thickening and of echotextural changes and fascicular patterns in various neuropathies. These features can be quantified objectively by measuring the CSA of the nerve. An increased CSA of the nerve involved permits a precise localization in compressive neuropathies and in neural tumors (25, 26).

The more severe consequences of leprosy such as deformities and disability are due to neurologic involvement (11). For this reason, the WHO suggests more studies with peripheral nerve ultrasound in neuropathy of leprosy since palpation of peripheral nerves is subjective and requires training (13), with agreement between pairs of professionals trained in the technique of nerve palpation being unsatisfactory (27), while HRUS has better cost-effectiveness than other methods, such as magnetic resonance (15, 28).

In the present study, the age range of 15–60 years was selected because it shows lower variation of absolute CSA values (mm^2^) of peripheral nerves (29), with lower values among children and adolescents and higher values among older individuals. Another important factor for this choice was the epidemiological distribution based on the time of disease incubation and risk of transmission, proposed for HD by dividing the age ranges into lower and higher than 15 years.

Our study stands out by involving the largest series in the world for the assessment of the peripheral nerves of patients with leprosy, with all examinations being performed by the same physician specialized in imaging diagnosis during an active search within the community and not solely within hospitals and reference centers.

The assessment of the differences in the CSA of the neural sites (ΔCSA and ΔTPT) distributed according to age range (Figure 4) demonstrated that the youngest has shorter time of disease and, consequently, lower differences between sides (ΔCSA) and between sites of the same nerve (ΔTPT). Only the ulnar nerve did not show change in the ΔCSA and ΔTPT with age, highlighting as an important nerve for the assessment of HD neuropathy.

For all neural sites, patients with HD presented higher absolute CSA values than healthy individuals (*p* < 0.0001), confirming the data reported by Frade et al. (10) in a study conducted at a reference center for HD.

**Figure 10:**
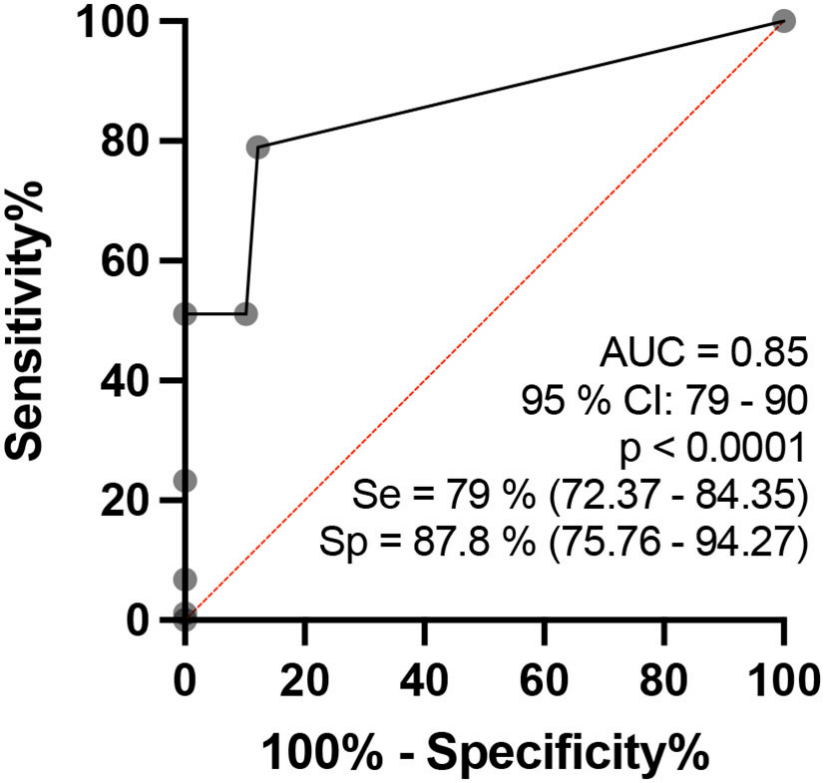
ROC curve analysis of the percentages of the number of asymmetries of the neural sites assessed per individual, which is considered to be altered (ΔCSA>2.5 mm^2^) when comparing the healthy and HD groups in the age range of 15 to 60 years.

Regarding asymmetry between the right and left sides, patients with HD also had higher ΔCSA values than healthy individuals (Figure 10), which is also in agreement with the data reported by Frade et al. (10).

**Figure 11:**
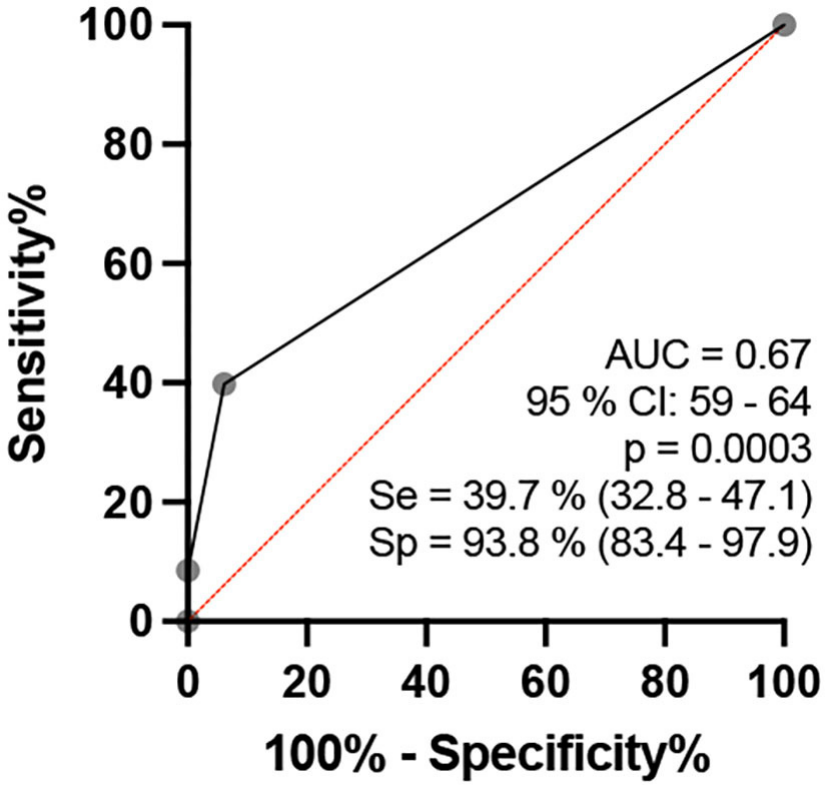
ROC curve analysis of the percentage of the number of altered ΔTpT (>2.5 mm^2^ - focality) of the neural sites assessed per individual when comparing the healthy and leprosy groups in the age range of 15–60 years.

Regarding focality, that is, the difference between two sites on the same nerve (the ulnar nerve), the highest mean, standard deviation, and median ΔTPT values were obtained for the HD group (HD: 2.3 ± 3.9 mm^2^/HV: 1.0 ± 0.8 mm^2^; median 1.0 mm^2^) (Figure 11). These changes characterize the neuropathy of HD as hypertrophic, asymmetrical, and focal, findings which confirm the data reported by Frade et al. (10) and Pottecher et al. (30).

Klauser et al. (31), Klauser et al. (32), and Miyamoto et al. (33) have reported that thickening of the median nerve in cases confirmed as having carpal tunnel syndrome occurs inside the tunnel. Nagappa et al. (34) recently showed that the thickening 2 cm proximal of the carpal tunnel for the median nerve discriminates leprosy from the carpal tunnel syndrome (34). The localization of neural thickening, such as in the ulnar nerve in the cubital pretunnel, helps in the diagnosis of leprosy neuropathy (10, 15, 19, 20, 35).

Jain et al. (20) detected at least one thickened nerve in 18/20 (90%) patients with leprosy and concluded that neural involvement affects a larger number of nerves and various neural sites in leprosy. Considering nerves greater than the upper normal limits to be altered (mean ± 2 SD), our data identified altered CSAs in 157/176 (89%) cases, in agreement with Jain et al. (20). In addition, 113 patients (64%) had altered absolute CSA values in at least three sites of peripheral nerves, a fact that defines leprosy neuropathy as hypertrophic.

Several laboratory tests have been studied for the diagnosis of leprosy, such as the use of anti-PGL1, which showed lower sensitivity than other antigens but did not affect specificity, and a meta-analysis showed mean sensitivity of 59.1% (95% CI 50.6–671) and specificity of 91.7% (95% CI 83.9–94.9). In 78 studies, ELISA was the test predominantly investigated among all available serological tests, with sensitivity ranging widely from 0 to 100% and specificity ranging from 13 to 100% (36).

Peripheral nerve thickening viewed by HRUS showed superior sensitivity and specificity for the diagnosis of HD neuropathy. Thus, if an individual has at least two neural sites (16.5%) defined as thickened, this finding has 76.1% sensitivity and 87.8% specificity, with an RR of 24. When at least one neural site (10%) of the nerves assessed shows asymmetrical thickening, sensitivity is 72.4% and specificity is 87.8%, with an RR of 13. Regarding the ΔTPT of the ulnar nerve lesion, 40% of the patients had at least one nerve, with a difference of more than 2.6 mm^2^, 39.7% sensitivity, 100% specificity, and an RR of 6.4 times.

Also, the authors bring some tips about when screening the patients:

- El Gency et al. (12) concluded that “a peripheral neuropathy accompanied by neural thickening with or without cutaneous manifestations should lead the clinician to think about HD,” so we indicate that for patients without sensory loss classified as glove or boot who present islands of sensitivity alteration, the clinician could perform ultrasound of peripheral nerves following the protocol for leprosy.
- Every individual with clinical suspicion of leprosy plus the clinical suspicion of neuritis (pain or shock in the nerve path) should be evaluated by high-resolution ultrasound following the protocol for leprosy.
- Every individual who is in contact with a patient with leprosy should be scanned by HRUS in order to carry out future follow-ups so that we can make an early diagnosis when there is nerve thickening associated with some functional loss.

There are some limitations to the study: All patients with HD were not assessed for the Med FA (median forearm) and CFTH (common fibular thigh), we did not compare these two neural points between HD and HV groups, and our data did not compare patients with HD with other neuropathies. Other limitations may be the use of three different machines, which may have some difference in relation to the CSA obtained on different machines, but we tried to strictly follow the protocols for measuring the CSA, which makes the study quite robust. All our data collection was carried out during active patient search campaigns in four Brazilian states; therefore, it was not carried out in referral hospitals. Therefore, we assume that these patients had a shorter time of disease or infection, perhaps even early; however, it is very difficult to answer this question exactly since the pathophysiology and natural history of the disease in patients with leprosy 2–5 years, according to some authors.

## Conclusion

HD assessed by HRUS is established as a primary neural disease that leads to multiple hypertrophic mononeuropathy characterized by CSA values exceeding normal limits: Med CT = 10.2 mm^2^; UT = 9.8 mm^2^; UPT = 9.3 mm^2^; CFFH = 18.3 mm^2^; T = 9.6 mm^2^.

An individual with at least two thickened nerves assessed in the active search campaign has a 23.1 greater chance of having leprosy than a healthy individual.

HD neuropathy is characterized not only by an increased CSA but also by the pattern of asymmetry (ΔCSA>2.5 mm^2^ with an RR of 13) and focality (ΔTPT>2.5 mm^2^ with an RR of 6.4) of this thickening, with high sensitivity and specificity for its early diagnosis.

Peripheral nerve ultrasound based on the protocol for the assessment of leprosy neuropathy (Med CT, UT, UPT, CFFH, and T nerves) can be used as a point-of-care method for the early diagnosis of HD neuropathy.

## Data availability statement

The raw data supporting the conclusions of this article will be made available by the authors, without undue reservation.

## Ethics statement

The studies involving human participants were reviewed and the present study was approved by the Research Ethics Committee of the University Hospital, Faculty of Medicine of Ribeirão Preto, University of São Paulo (protocol no. 2.165.032, MH-Brasil and 92228318.1.0000.5440). Written informed consent to participate in this study was provided by the participants' legal guardian/next of kin.

## Author contributions

GV and MF: contributed on all stage of this study, to conception and design of the study, performed the statistical analysis, to manuscript revision, read, and approved the submitted version. FF, ND, CSi, ML, JS, JB, CSa, GC, and MD: contributed to conception and design of the study, to data collection, obtaining, analyzing and interpreting data, and approved the submitted version. All authors contributed to the article and approved the submitted version.

## Funding

Coordenação de Aperfeiçoamento de Pessoal de Nível Superior - Brazil (CAPES) - Finance Code 001. National Council for Scientific and Technological Development (CNPq). Center of National Reference in Sanitary Dermatology and HD - Hospital of the Medical School of Ribeirão Preto, Ribeirão Preto, São Paulo, Brazil; the Brazilian Health Ministry (MS/FAEPAFMRP-USP: 749145/ 2010 and 767202/2011); Oswaldo Cruz Foundation (Fiocruz) Ribeirão Preto - TED 163/2019 - Process: No 25380.102201/2019-62/ Project Fiotec: PRES-009-FIO-20; VALE S.A. 27756/2019.

## Conflict of interest

The authors declare that the research was conducted in the absence of any commercial or financial relationships that could be construed as a potential conflict of interest.

## Publisher's note

All claims expressed in this article are solely those of the authors and do not necessarily represent those of their affiliated organizations, or those of the publisher, the editors and the reviewers. Any product that may be evaluated in this article, or claim that may be made by its manufacturer, is not guaranteed or endorsed by the publisher.
