# Supplementary material for: Point-of-care ultrasound of peripheral nerves in the diagnosis of Hansen's disease neuropathy
Source: Front Med (Lausanne). 2022 Sep 9;9:985252. doi: 10.3389/fmed.2022.985252 (PMC9504868; doi:10.3389/fmed.2022.985252)
Supplement: Supplementary file 1 [file Data_Sheet_1.pdf]

Annex 1:

Attached to this study, in order to facilitate the interpretation and comparison of data from patients diagnosed with leprosy with healthy individuals, we insert table 2 of the article “Ultrasonography reference values for peripheral nerve cross-sectional areas and indexes in a Brazilian sample” accepted for publication in the journal Radiologia Brasileira in July 2022. For cross-sectional study, we recruited 85 individuals without peripheral neuropathy at health units of the Southeast, North and Northeast Brazilian regions. Exclusion criteria were the identification of neurological symptoms (loss of strength, paresthesia, electric shock-like pain, pain, and cramps) or a body mass index above 35.0 kg/m2, metabolic diseases or peripheral neuropathy diagnosis, and history of limb amputation.

Table 1: Distribution of ultrasound measurements (CSA, ΔCSA, ΔTPT)) in healthy volunteers with 15-60 y and upper limit: mean ± standard deviation, median in square brackets.

**Variables**	Age Range (years)	15-60 years	Upper limit (mean+2SD)
	(n) Men	22	
	(n) Women	27	
	(n) total (R+L)	98	
	Mean±SD/Median	27±13[25]	
	**Sites**	**Mean ± SD [Median]**	
**CSA of peripheral nerves (mm^2)**	MT	6.6±1.9[6]	10.4
	UT	6.7±1.5[6.1]	9.7
	UPT	6.0±1.7[5.4]	9.4
	FH	10.7±3.8[9.9]	18.3

	MT	0.8±0.7[0.9]	2.2
**ΔCSA (mm^2)**	UT	0.9±0.6[0.9]	3.1
	UPT	0.6±0.4[0.5]	1.4
	FH	0.9±0.7[1]	2.3
**ΔTPT (mm^2)**	Ulnar (UT and UPT)	1.0±0.8[1]	2.6

Legend: n: number of individuals; R: right, L: left; MT: median nerve in the carpal tunnel; UT: ulnar nerve in the cubital tunnel; UPT: ulnar nerve proximal to the cubital tunnel; FH: common fibular nerve close to the fibular head; ΔCSA: difference between the largest and smallest CSA measurements; ΔTPT: the same nerve difference between the largest and smallest CSA measurements of PT and T of left plus right sides; CSA ratio: dividing the largest and smallest CSA measurements for each nerve point independent of the side; TPT ratio: the same side dividing the largest and smallest CSA measurements of PT and T points along the ulnar nerves; * = $p<0.05$ as compared to all other groups ranged by age.
