# Supplementary material for: Point-of-care ultrasound of peripheral nerves in the diagnosis of Hansen's disease neuropathy
Source: Front Med (Lausanne). 2022 Sep 9;9:985252. doi: 10.3389/fmed.2022.985252 (PMC9504868; doi:10.3389/fmed.2022.985252)

Annex 2

Attached 2 of this article is supplementary material with a board developed by the authors with the main morphological patterns observed during the data collection activities (data not yet published that could be used for future multicenter studies). And, there are other boards with several image captures and basic editing on peripheral nerves obtained in patients diagnosed with leprosy.

Plate 1: main morphological patterns of the cross-sectional areas seen by the high-resolution ultrasound observed by the authors.

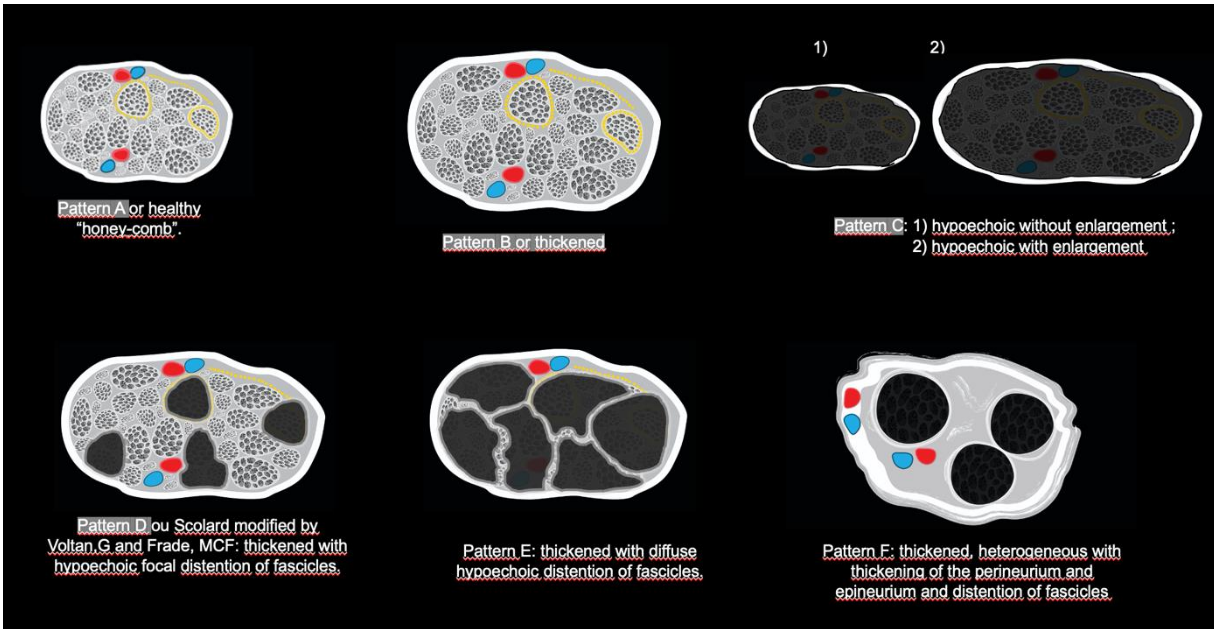

Plate 2: Figure 2 A-D: Cross section demonstrating CSA of the NFSup from distal to proximal in the ankle.

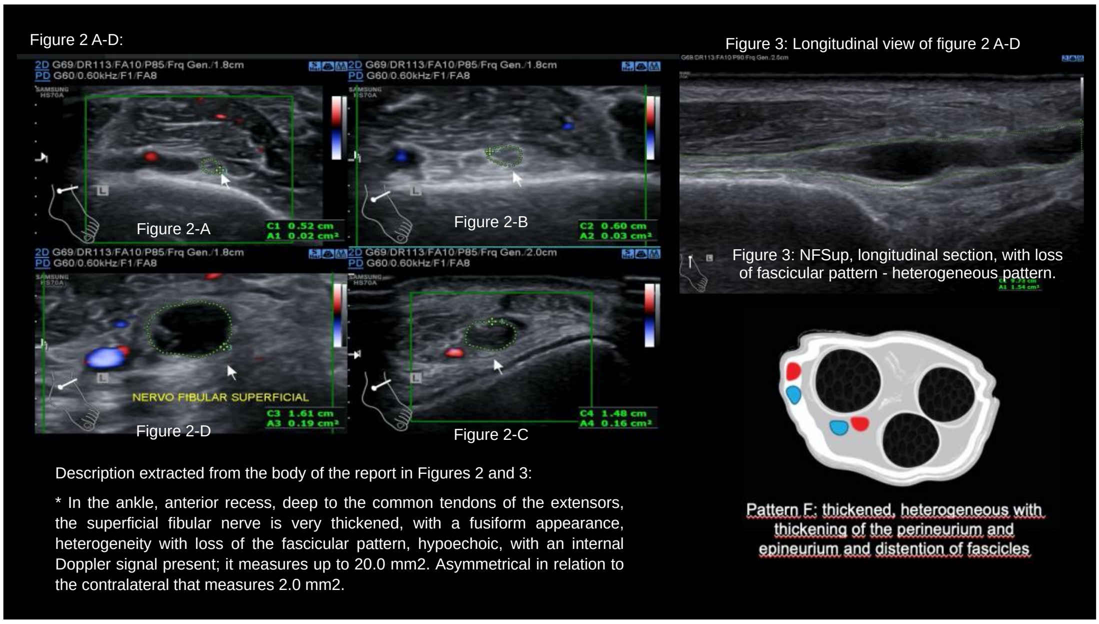

Plate 2: schematic draw of neural abcess (A); real image of ultrasound (B).

A

Schematic drawing from a high-resolution ultrasound image of a peripheral nerve abscess in longitudinal view with panoramic and positive Doppler signal.

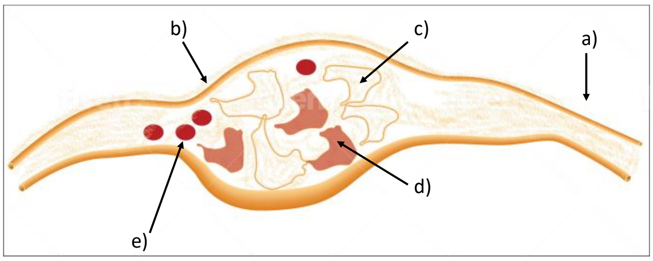

a) Nerve

b) Fusiform neural thickening: FOCUS

c) Signs of chronicity/Fibrosis: Heterogeneous hyperechoic areas – loss of the "honey-comb" pattern.

d) Small intraneural abscesses: anechoic areas with acoustic enhancement

e) Doppler dots

B

Neural pseudotumor – Untreated chronic neuropathy exacerbated after Covid-19 infection.

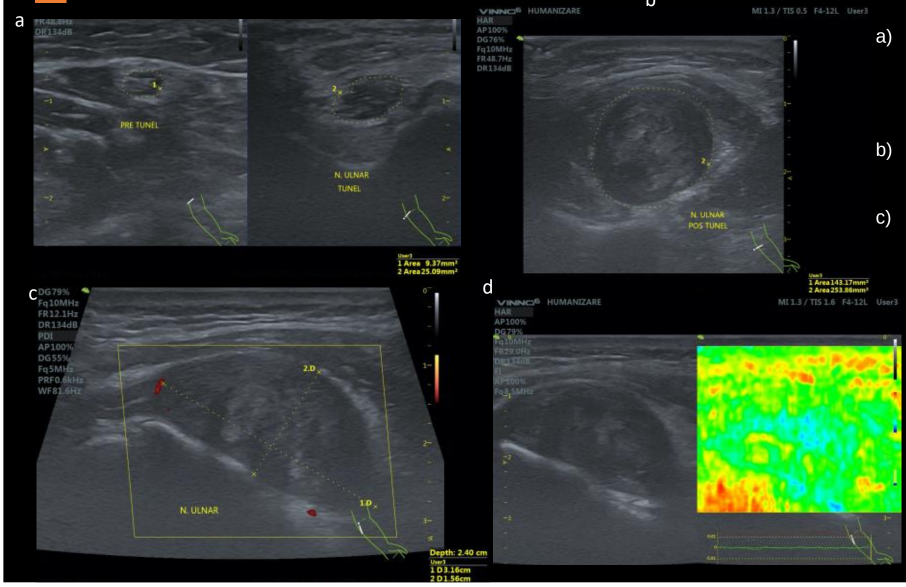

a) Ulnar nerve in transversal view on proximal of tunnel cubital and cubital tunnel with focal thickening;
b) ulnar nerve on post-cubital tunnel (pain point);
c) and d) nerve with fusiform focus, loss of fascicular pattern with hyperechoic areas with increased elastoscan hardness, and anechoic areas with low hardness intermingled, suggestive of microabscess.

Plate 4: Cutaneous branch of the musculocutaneous branch in the forearm: a) and b) are transversal and longitudinal images performed with usg up to 12 MHz – WE DO NOT CAPTURE DOPPLER; c) d) and e) are cross-sectional images made with 18-23 MHz usg – EXUBERANT DOPPLER.

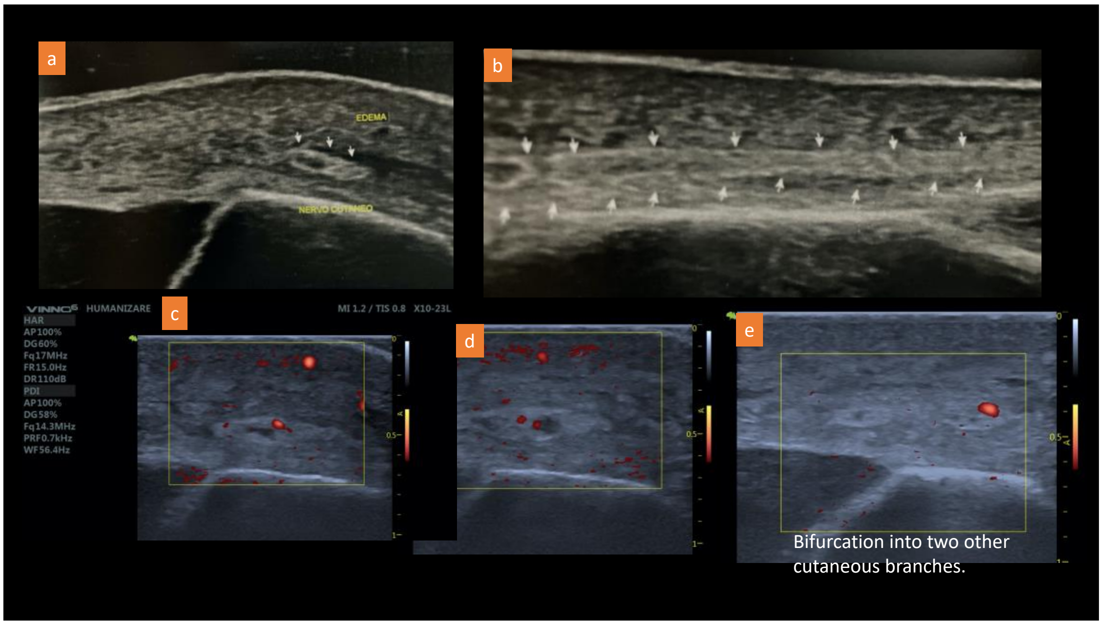

Plate 5: Cross-section of the ulnar nerve on diferente patients showing: increase in the cross-sectional area with a heterogeneous fascicular pattern with hypoechoic distension of the fascicles and diffuse thickening with increased echogenicity of the epineurium, suggesting chronicity and neural fibrosis.

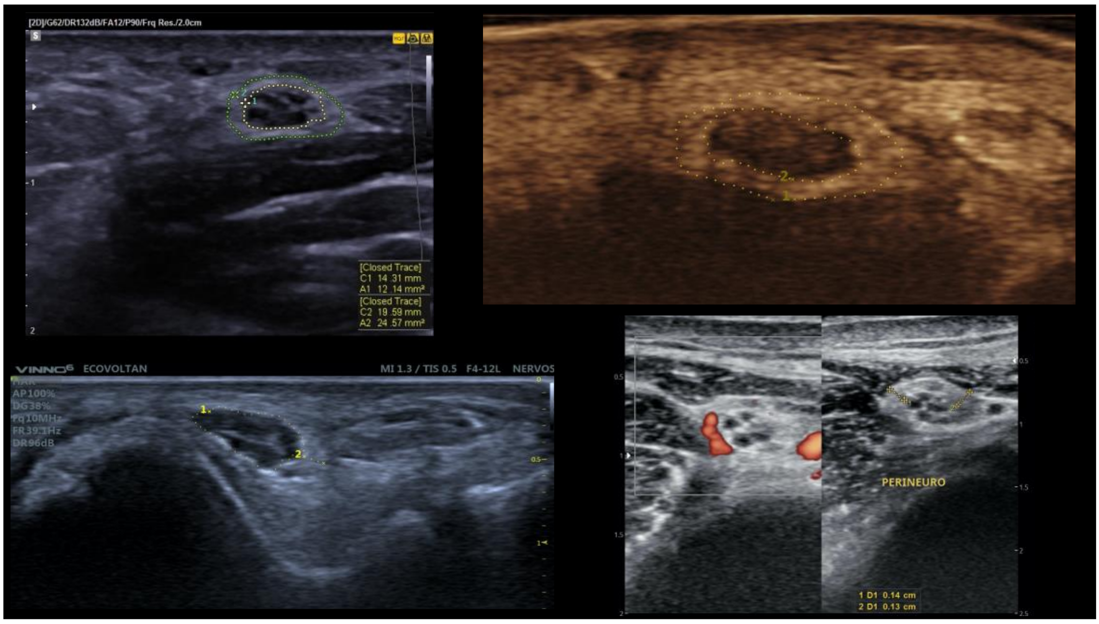

Supplement: Supplementary file 2 [file Data_Sheet_2.pdf]
